# Life in the Sick-Room. Essays by an Invalid

**Published:** 1844-10

**Authors:** 


### Art. XV.

*Life in the Sick-room. Essays by an Invalid.* — *London*, 1844. 12mo, pp. 221.

The pathologist thinks no description too minute to discriminate the shades of colour, the varieties of consistence, and the changes of form which the material textures of the body undergo in disease: but have the multiform varieties of morbidity of mind been subjected to a similarly systematic scrutiny? When this reaches insanity we recognize it as a disease coming within our province, but minor shades of mental infirmity we leave to theologians and moralists. And yet, though it may be convenient to divide diseases into smaller departments, the genus as a whole should not be lost sight of; for it is alike important to recognize the individual members of a family, and their relationship and mutual dependence as a whole. And as all deviations from a healthy state of mind belong to that family of which the most marked condition is insanity, all shades of unsoundness of mind should be included in a comprehensive survey of this disease, and should demand our attention. The improved treatment of insanity in the present day has been owing to our recognition of it as a bodily disease, as well as a mental one, and instead of treating those who are affected with it as beings possessed with devils, and only to be managed by chains and dungeons, bringing them into the same class as those whom we admit to be objects for our hospitals. We are still much in the dark as to the connexion between insanity and organic disease, but we are pursuing the right path. And does not the same argument apply to minor shades of mental disorder? We must be very inattentive observers not hourly to recognize that a very intimate relation does exist between morbid states of the body and minor diseases of the mind—ill-temper, ill-nature, gloom, high spirits, moroseness, fickleness, confidence, despair, passion, and desire; and the due recognition of this fact is very essential. When not duly recognized, and the affection is regarded as a mere spiritual condition, the mistakes committed are of the same kind as in insanity, and less injurious only because the disease was less important. How much good advice, and wise cautions and admonitions and recommendations of good books have not been thrown away, when perhaps the physical remedy which should have preceded the moral one was neglected! Viewed in this light, all varieties of unsoundness of mind are a subject of great interest, as the knowledge of their connexion with diseased bodily states may direct us to the restoration of a healthy mental condition, by assisting moral remedies with physical ones. In another point of view the study of mere mental conditions is important. They do exist—they are realities to which our patients are very subject, and of which we not unfrequently feel the inconvenience; and therefore it behoves us to be acquainted with them.

These are our reasons and excuses for directing attention to a work, not professedly medical, but which contains the revelations, to a certain extent, of the morbidity of mind of a nervous invalid.

We gather from the internal evidence of the book itself that the writer is a confirmed invalid, confined to her room, and convinced that she is "under sentence of disease for life." She is a constant sufferer from pain, sometimes of the acutest kind, and the habitual use of opium has become an invariable habit. Debility of body, languor, malaise, incapacity for continuous exertion, with loss of all gaiety of mind and sense of enjoyment; and, with the exception of a few hours twice in a year of light-heartedness, over all the rest a cloud of care—apparently causeless, but not the less real;—are the bodily and mental symptoms which in her case, as in so many of her sex, indicate an exhausted nervous system.

But a very few lines in her book, scattered here and there, are devoted to a description of her bodily ailments; the object of the writer is to unfold the effects of sickness upon the mind, and its manifold helps and hinderances, perils and pains, advantages and compensations, injury and profit: a comprehensive theme, well worthy of deep study, and one which can be treated of satisfactorily alone by one who has had a long apprenticeship to suffering. This book differs materially from the majority which fall under our notice as critics. Where every word tells it is impossible to condense, and where each sentence is full of matter, and all closely connected into a whole, it is difficult to abridge. It is not our province to go into the whole subject: we shall therefore confine ourselves to those parts only which have some practical bearing, either illustrating morbid states of mind, the existence of which or their connexion with disease of body in invalids it is our province to recognize and to study, or affording hints for the better management of such patients. Those who are further interested in the subject, and who are desirous to know the uses to be made of adversity, we must refer to the book itself.

To man, with his stronger self-reliance, the deep need which women have of sympathy is a matter of observation rather than of experience, and he cannot fully enter into the want. It has been said that man lives in his understanding, woman in her affections; and if so, the fellow feeling which a cultivated man needs is more with his intellect than his feelings. But as the feelings are much more connected with bodily states, the sympathies with feelings which women seem absolutely to require, must necessarily be very considerably influenced by the disorders of the body; and no part of this volume is more strikingly morbid than its revelation of sympathies. There is a separate chapter on sympathy; but the intense craving after this, which is called "a hunger of the heart," "a heavenly solace," and the delight which those afford who have the "true genius for sympathy," who are "archangels of consolation," and the uneasiness, irritability, and vexation which awkward consolers give, are disclosed throughout the book. The following hint to "awkward sympathisers" may be remembered when dealing with this class of patients:

"Going back to the days when I, myself, was the sympathiser, I remember how strong is the temptation to imagine, and to assure the sick one, that his pain will not last; that the time will come when he will be well again; that he is already better; or, if it be impossible to say that, that he will get used to his affliction, and find it more endurable. How was it that I did not see that such offers of consolation must be purely irritating to one who was not feeling better, nor believing that he should ever be better, nor in a state to be cheered by any speculation, as to whether his pain would, or would not, become more endurable with time! Exactly in proportion to the zeal with which such considerations were pressed, must have been the sufferer's clearness of perception of the disguised selfishness which dictated the topics and the words. I was trying to console myself, and not my friend; indulging my own cowardice, my own shrinking from a painful truth, at the expense of the feelings of the sufferer for whom my heart was aching." (p. 14.)

It is difficult for men in health to realize the evident annoyance which such invalids feel at any hope being held out of recovery, but the physician often meets with such. Is it not from the deeper sympathy which is seemingly given, if the cases are considered hopeless? The "genius for consolation" does this.

After expressing her irritability at one of the well-meaning, who had no tact, she adds,

"Far different was my emotion, when one said to me, with a face like the face of an angel, 'Why should we be bent upon your being better, and make up a bright prospect for you? I see no brightness in it; and the time seems past for expecting you ever to be well.' How my spirits rose in a moment at this recognition of the truth!" (p. 15.)

Was it the mere announcement of a truth which caused this exhilaration? Was it not rather the deeper knowledge which this lady-friend had of the invalid's feelings, and of her "keen hunger of the heart," which enabled her to fathom her wishes, and to give just that quantity of the desired food which she craved? Is not this view borne out by this subsequent resolution?

"Never again should the suffering spirit turn from me, as I fear it has often done,—if too gentle to be irritated—yet sickening at hollow words of promise, *when instant fellow-feeling was what was needed.*" (p. 18.)

A man of the highest intellectual power, who was himself an invalid, a severe sufferer from pain, to ease which he habitually took opium, said "that the best physician in nervous diseases was the best inspirer of hope." This was a man's feeling. He resents pity; but how different with a woman; with her "pity is akin to love;" give her no hope and she blesses you.

But how is it possible to soothe the sufferer?

"Speaking the truth in love, is the way. Then the question arises, what sort of truth? Why, that which is appropriate to the one who administers. Let the nurse avow that the medicine is nauseous. Let the physician declare that the treatment will be painful. Let sister, or brother, or friend, tell me that I must never look to be well. When the time approaches that I am to die, let me be told that I am to die, and when. If I encroach thoughtlessly on the time or strength of those about me, let me be reminded; if selfishly, let me be remonstrated with. Thus, to speak the truth in love, is in the power of all." (pp. 26-7.)

The whole chapter will repay perusal. The genial healthy reader will become acquainted with states of feeling which are new to him; the more sickly mind will find some of his own feelings embodied in words, and both will learn something as to the best way of dealing with such patients.

What should be the arrangements of a permanent invalid, with regard to companionship, is an important consideration.

"In most cases, this is no matter of choice, but a point settled by domestic circumstances; where it is not, however, I cannot but wish that more consideration was given to the comfort of being alone in illness. This is so far from being understood, that, though the cases are numerous of sufferers who prefer, and earnestly endeavour to procure solitude, they are, if not resisted, wondered at, and humoured for a supposed peculiarity, rather than seem to be reasonable; whereas, if they are listened to, as the best judges of their own comforts, it may be found that they have reason on their side. (p. 30.) There is no attendance to be compared with that of a servant." (p. 34.)

This unsentimental, but very practical truth, our invalid accounts for metaphysically, and dresses up her reasons becomingly and sentimentally. But many of the reasons with which the work abounds are evidently à posteriori arguments. The matter is first settled, not by any argumentative process whatever, but by the will, and then the decision is accounted for in the most "interesting" way. But, although "sic volo, sic jubeo" is the feminine way of settling such matters, she is not disposed to add "stet pro ratione voluntas," but rather to puzzle herself afterwards, with trying to frame reasons; a mistake; as she often arrives at the truth in such practical matters by a shorter and surer process than ratiocination.

"When an invalid is under sentence of disease for life, it becomes a duty of first-rate importance to select a proper place of abode. Many a sufferer languishes amidst street noises, or passes year after year in a room whose windows command dead walls or paved courts.

"He will be wise to sacrifice indolence, habit, money, and convenience, at the outset, to place himself where he can command the widest or the most beautiful view that can be had without sacrificing advantages more essential still. There are few things more essential still; but there are some: such as medical attendance, and a command of the ordinary conveniences of life."

Our invalid has chosen a sea view, for its variety, and from the wide expanse of sky it affords. But not too much sea, which would tire; between her house and the sea is a green down, a river, ponds, the ruins of a priory, and beyond a busy harbour; a sandy beach, and a rocky shore. A windmill, a railroad, a colliery, farm-yards and paddocks give a living interest to the whole, and here a telescope is a great additional source of pleasure. This whole chapter, entitled "nature to the invalid," is genial and beautiful. To such invalids, flowers are the most acceptable presents; and "fern-houses" (that is, ferns growing in close glass-cases, devised by a member of our profession) are in a town "a compensation for rural pleasures," and in the country an addition to them.

The good effects of books of travels are alluded to. Such are advisable from carrying the invalid out of himself for a time; they are objective, whilst his mental malady is "subjectivity." The effect of seclusion, in rendering the mind speculative rather than practical, is discussed. (p. 98.) The writer thinks that large and more distant views are taken, and that many doubts and difficulties which beset the mind immersed in actual life, are cleared away: that "moral considerations are all in all," and that seclusion is peculiarly fitted for seeing their beauty and abundance, and recognizing the "deep heaven lying inclosed in the very centre of society, and a genuine divinity residing in the heart of every member of it."

"All that is most frivolous and insignificant is ever most noisy and obtrusive; all that is most wicked is most boastful and audacious; all that is worst in men and society, has a tendency to come uppermost; and thus, the most superficial observers of life are the most despondent. Meantime, whatever is holy, pure, and peaceable, works silently and unremittingly; and while turbulent passions are exhausting themselves before the eyes of men, a calm and perpetual renovation is spreading outwards from the central heart of humanity." (p. 101.)

A chapter full of matter worth reflecting on is devoted to "temper." It was John Hunter, we think, who used to define organic irritability as action with weakness. And all forms of "temper," or mental irritability may be included in the same definition. Two forms are mentioned,—dissatisfaction with others, and dissatisfaction with one's self; they are opposite poles of the same mental disease, but they alike render their possessor miserable to himself, and an object either of pity, annoyance, or dislike to those who come in contact with him.

"Ill-temper" (so-called) or dissatisfaction with others, is too well known to be dwelt upon, we shall merely quote the following judicious hint;

"It is clear to the idlest and most selfish mind, that the whole hope of comfort in the sick-room, depends on the freedom and cheerfulness of the intercourse held in it,—a freedom and cheerfulness forfeited by irritability on the part of the sufferer, necessarily forfeited even if he were tended by the hands of angels." (p. 128.)

But we pass on to that other form, "dissatisfaction with one's self," and this is often the sore malady of those who never "feel the slightest inclination to be cross."

"This lowering, depraving tendency to self-contempt, requires for its establishment as a fault of temper, long protraction or permanence of illness; but when once established, it is as serious a fault of temper as can be entertained. Where religious faith and trust are insufficient for the need, this temper is almost a necessary consequence of any degree of mental and moral activity in a sick prisoner."

The injurious effect of this condition on the individual, as well as on those he comes in contact with, are thus acutely analysed:

"Without self-respect, there can be none of that healthy freedom of spirit which animates others to freedom, and exerts that influence which is ascribed to 'a good temper,' which removes hesitancy from the transactions of the daily business of life, and so permits life to appear in its natural aspect. Instead of this, where the spirit has lost its security of innocence, unconsciousness, or self-reliance, and become morbidly sensitive to failures, and dangers, where it has become cowardly in conscience, shrinking from all moral enterprise, and dreading moral injury from every occurrence, the temper of anxiety must spread from the sufferer to all about him, whether the causes of his trouble are intelligible to them or not. This abasement may coexist with the most perfect sweetness, and gentleness of speech and manners, and the sufferer may enjoy great credit for not being irritable, when he is in a far lower state than often coexists with irritability." (p. 131.)

This masterly dissection of one form of morbid mind, gives to the reader that vivid impression of truth which no writer on such subjects can produce, unless he writes out of his own heart. What is the remedy? We believe that this condition is much connected with an exhausted state of the nervous system; it is one of those forms of disease which result from the over-excitement of the nervous system continued so long as to be followed by permanent depression. Our neighbours alone, (notwithstanding, or perhaps owing to their natural gaiety,) have words expressive of other shades of this unhappy condition, *ennui*, *blasé*, or still more exquisitely discriminative the epithet which Madame Maintenon applied to Louis XIV, when she complained that she had to amuse a king who was no longer "*amusable.*" To ascertain the cause when it is a physical one, as it often is, is the first step towards a cure. But in a case like the one here supposed, of incurable (or rather we would hope uncured) indisposition, in which the patient is confined to her room, and indebted to opium alone for physical ease, a moral remedy can alone be offered, and the following advice is the resnlt of our invalid's personal experience :

"To revive healthy old associations, to occupy the morbid powers with objects from without, and to use the happiest rather than the lowest seasons for leading the mind to a consideration of its highest relations." (134.) "The appeal must be in seasons of ease and enjoyment, to the sense of dependence on God; and, in times of mental distress, to the principles of endurance and self-mastery." (p. 134.)

We have said that we believe the physical cause of this condition may be an exhausted nervous system. The causes of this may be physical or mental, or more often both. It may be the result of the early and long-continued indulgence in sensual excitements; or of the wear and tear of mind by its intense application to business or to study, with great neglect of the health. Disappointment in women of a very sensitive temperament is a frequent cause. But looking at it psychologically, is it not a form of pride, or rather of morbid vanity? Extremes meet. There is a pride which apes humility, and this too unconsciously. Dissatisfaction implies that the mind is annoyed at and resents its own low condition. True humility would acknowledge that there was tco much reason for this, and would wisely submit. And if the root of this defect is pride and vanity, then unconditional submission is the cure. What are "appeals to endurance and self-mastery," but deceitful and injurious flattery, which, like opium, relieves pain for the time, at the expense of aggravating and fixing the disease. The hardest lesson in the world to learn is that of humility, more difficult than the endurance and self-mastery of the hardest and coldest stoicism. Christianity alone teaches its importance and the sole means of its attainment.

Another "temper of the sick-room" is "particularity about trifles:"

"This, though often reaching a point of absurdity, should be scrupulously indulged, because no one but the sufferer can be fully aware of the annoyance of want of order in so confined a space and range of objects. A healthy person, who can go everywhere at pleasure, leaving litters to be put away by servants during absence, can have no idea of the oppression felt by a feeble invalid, when looking round upon the confusion left in one little room by careless visitors, chairs standing in all directions, books thrown down here and there, and work or papers strewed on the floor." (p. 134.)

We quite agree with our invalid that this particularity should be indulged, as although to a healthy mind it is incomprehensible and absurd, yet it is a source of real annoyance to the nervous invalid, and on this account it should be remembered in "the management of the sick-rooms" of such patients.

"No one challenges this particularity when it relates to hours. The most careless observer must know that it is illness of itself to a sick person, to have to wait for food, or medicines, or to be put off from regular sleep." (p. 135.)

We are all, when in health, apt enough to "dismiss our uneasy sympathies" by assuming that invalids become inured to suffering, and to moralize on this wise provision. But how few of us take into consideration the meaning of this cheap phrase, "becoming inured," and reflect "on the series of keen mortifications, of bitter privations" which it includes—even in those cases in which the inuring process at last brings relief! But there are sufferings to which this slow relief never comes. Those who have watched the progress of tic douloureux, stone, carcinomatous tumours involving nerves, or cancer of the uterus, will assent to the individual experience of this writer.

"To almost every kind and to vast degrees of privation—moral and physical—men may become inured; and to chronic sufferings of mind and body: but I am convinced that there is no more possibility of becoming inured to acute agony of body than to paroxysms of remorse—the severest of moral pains. The pain itself becomes more odious, more oppressive, more feared, in proportion to the accumulation of experience of weary hours, in proportion to the aggregate of painful associations which every visitation revives... For the sake of both sufferers and sympathisers it would be well that this should be thoroughly understood, that aid may not fall short, nor relief be looked for in the wrong direction." (pp. 147-8.)

We have thus given the Invalid's own description of some states of mind and feeling produced by permanent and long-continued disease of body. We must refer to the volume itself for the consolations offered, and for the writer's own views on the compensations afforded to such withdrawal from active life, by increasing the power of that prerogative of a thinking being, "looking before and after." But there is another side from which this book may be studied: not merely the writer's own conscious conception of her state of mind, but the display of her own actual condition which every such writer makes, to a great extent unconsciously.

That the writer is a woman would, we think, have been evident from the work itself, had it not been attributed, without contradiction, to one of the best known authoresses of the day. The confession of such faults and failings as create "interest," the hunger after sympathy, the undisguised love of praise, the annoyance at "not being understood," are unmistakable characteristics. Admitting, as we do most fully, the writer's intellectual powers, her lofty and deep thoughts conveyed in a style of consummate clearness, a true feeling for the beautiful and the good, and the power of embodying the poetry of her mind in graceful, captivating prose, aspirations after a high state of moral purity, and a deep conviction that man's moral being should be his prime care—yet we cannot help feeling—and all the stronger, perhaps, for our favorable impressions—to quote the saying of a child to herself, which she repeats more than once: "But there is unhealthiness, and that spoils all."

The head desires ardently to strike the stars; but the poor unwholesome body pulls it down very low, and contradicts its right to any such elevation. The contradiction between the idealism of the abstracted mind, and the actual feelings of that mind when brought into contact with realities, affords the strangest and strongest contrasts. The world and its great parties are seen in a new and clearer light: they are looked at by the invalid with stoical philosophy, as parts of history: what before was dark in the ways of Providence, becomes clear: the common distinctions of rank are "attenuated to a previously inconceivable degree," so that "there would be little more in the sovereign entering the sick-room than any other stranger whom kindness might bring"—"so overpowering in our view are the great interests of life which are common to all—duty, thought, love, joy, sorrow, and death." And yet—alas for poor human nature—if a kind visitor, who has not the organ of order well developed, leaves the chairs awry, and the books unreturned to their shelves, all this lofty philosophy is overthrown, and its possessor cast into a state of miserable irritability. With the clearest supposed insight into the most difficult problems that politicians are working out, there is an incapacity to appreciate correctly the individual's own condition, producing unreasonable dissatisfaction with self, self-distrust, even self-disgust. With the conviction of the paramount importance of being, rather than seeming, there is the painful wincing at a suspicion of being misinterpreted, and not understood—at being thought to be more comfortable, or more miserable as an invalid than in health; at any outward circumstances, in fact, which contradicts at the moment the peculiar feeling of the mind. We do not bring this forward as mere criticism, but as truth in which we are deeply interested as pathologists, as elucidating the effects of disease of body on the mind ; and the conclusions we would draw from it are :

1. That the feelings which are certainly most closely connected with the body are the most affected by this disease in which the nervous system is exhausted and depressed, and that they are the most morbid ; whereas the intellect remains little disturbed, and, when able to act at all, capable of clear thought.

2. That the advice offered to the individual by his own intellect is very incapable of correcting these disordered states of the feelings.

3. That the mere intellectual powers may be cultivated and improved to a very high extent, and yet, if there is depressing disease of the nervous system, the feelings and what are called the active powers which put us in relation with our fellows, may be in a much lower state than in the healthy whose intellects are comparatively uncultivated—simple-hearted, good people, who are engaged in the active and humblest duties of life.

Self-consciousness has been said to be the disease of the present day ; and assuredly a morbid self-consciousness which defeats its object, and becomes blind and puzzled, and bewildered by dwelling too incessantly on the individual's emotions and feelings is one of the morbidities of mind portrayed here. A celebrated thinker of the present day goes so far as to assert that all self-consciousness is disease, that the mind should no more be conscious of its operations than the stomach, and that in either case the consciousness of the work going on, is disease. We cannot go this length. The power by which the mind observes its own acts and judges them as if there were two beings within a man, one the passive spectator of the other's thoughts, feelings, and actions, is the prerogative of the highest class of minds. Much of what we call genius consists in the power of embodying these observations which the soul makes on herself, in words, "giving to airy nothings a local habitation and a name," so that others less highly gifted with expression, on reading them embodied in words exclaim, "I have thought and felt the same thing." "This comes home to me." "This meets me." And although all have not this genius, yet the power of looking inwards is common to all who think at all. It cannot then be called a disease, and be put in the same class with gastrodynia, hypochondriasis, or palpitations of the heart, without a very false and hasty generalization. But, like all other natural powers, it may become diseased when too exclusively cultivated. It becomes a lamentable disease, especially when the feelings and emotions are too constantly self-contemplated, and one to which cultivated minds are peculiarly liable. Its corrective is action; and when invalidism prevents this wholesome counterbalance, a sensitive and thoughtful mind is very apt to become engrossed in its own thoughts and feelings, and to get into a condition of helpless and egotistical selfishness; just in the same degree as a mere hypochondriac becomes engrossed in his own bodily sensations. As we can know our own powers by action only, this condition of self-consciousness is (although seemingly a contradiction) one of great self-ignorance. Whatever becomes the object of constant thought or attention becomes unduly magnified. The individual's own importance is thus greatly increased in his own estimation, and when called into action or into collision with others, the consciousness of his weakness, (often true from want of practice,) combined with his own sense of self-importance makes him irritable to others, or dissatisfied with himself, or both. This unreasonable dissatisfaction with one's self, this "mental weakness which converts the most innocent and ordinary occurrences into occasions of apprehension, or of self-distrust, or self-disgust," is one to which the weaker sex are especially liable, and particularly those who, though compelled to inaction by disease, have active intellects, and quick and strong feelings.

The mode in which self-consciousness becomes a disease, although in itself a healthy and natural mental act, may be explained on known physiological and pathological principles.

The functions of all parts increase by use. By directing attention to an organ under the power of the will an increased supply of blood and of nervous influence is sent there, and the continued action of the same cause in a healthy body may make this condition permanent, or in an unhealthy one, it may produce congestion, with temporary excitement followed by corresponding depression. As the mind acts through bodily organs, when we direct our own attention to our own inward mental feelings or emotions, we are in fact directing it to the material organs of those emotions. We are directing our attention to certain parts of the nervous system, or of the internal bodily organs, and stimulating them. The effect of this in a weak organ may be to increase its powers for a time, by sending it a large supply of blood, and to be followed by collapse and debility, as the reaction from over-excitement. And as we know of other organs, the weaker they really become the more prone they may be to temporary congestions of blood or nervous power, and thus get into a permanently irritable condition,—action with weakness; so, this state at last gets beyond the power of the will which first may have produced it, by directing the attention to it.

And this in conclusion leads us to the importance of a healthy state of body. "Non sine animo corpus, nec sine corpore animus bene valere potest."

It is the full and free recognition and belief that we consist of mind and body; that the mind is the great spiritual power moving the body; the dynamic force making matter its agent in acting upon the outward world, and that in order to exert its full power, it must have a material agent fully capable of obeying its dictates, a perfect machine at its command,—which will lead us to attend to the bodily health as well as the mental, in all stages of our growth and education. And in this way an example of a large class in which the mind has been attended to, and most highly cultivated, and the body neglected, is a useful lesson. We cannot give more force to this conviction than by putting the mind here indicated in contrast with one in whom both body and mind were freely and fairly developed, the healthiest literary man both in body and in mind of our time, drawn by the most forcible and original British writer of the present day:

"Scott was a genuine man, which itself is a great matter; no affectation, fantasticality or distortion dwelt in him, no shadow of cant. Nay withal, was he not a right brave and strong man, according to his kind? What a load of toil, what a measure of felicity he quietly bore along with him; with what quiet strength he both worked on this earth and enjoyed it, invincible to evil fortune and to good! A most composed invincible man: in difficulty and in distress knowing no discouragement, Sampson-like, carrying off on his strong Sampson-shoulders the gates that would imprison him; in danger and menace laughing at the whisper of fear. And then, with such a sunny current of true humour and humanity, a free joyful sympathy with so many things. The truth is, our best definition of Scott were perhaps even this, that he was, if no great man, then, something much pleasanter to be, a robust, thoroughly healthy, and withal, very prosperous and victorious man. An eminently well-conditioned man, healthy in body, healthy in soul; we will call him one of the healthiest of men. Neither is this a small matter: health is a great matter, both to the possessor of it and to others. A healthy body is good, but a soul in right health is the thing beyond others to be prayed for, the blessedest thing this earth receives of heaven. Without artificial-medicament of philosophy, or tightlacing of creeds, the healthy soul discerns what is good, and adheres to it, and retains it,—discerns what is bad, and spontaneously casts it off. In the harmonious adjustment and play of all the faculties, the just balance of one's-self gives a just feeling towards all men and all things. Glad light from within radiates outwards, and enlightens and embellishes. Now all this can be predicated of Walter Scott, and of no British literary man that we remember in these days to any such extent, if it be not perhaps of one, the most opposite imaginable to Scott, but his equal in this quality, and what holds of it,—William Cobbett—Cobbett as the pattern John Bull of his century; strong as the rhinoceros, and with singular humanities and genialities shining through his thick skin, is a most brave phenomenon. So bounteous was nature to us; in the sickliest of recorded ages when British literature lay all puking and sprawling in Werterism, Byronism, and other sentimentalism, nature was kind enough to send us two healthy men. A healthy nature *may* or not be great, but there is no great nature that is not healthy." (Thomas Carlyle.)

---

### Art. XVI.

*Recherches et Observations sur les Causes des Maladies Scrofuleuses.* Par J. G. A. Lugol, Médecin de l'Hôpital Saint-Louis, &c.—*Paris*, 1844.

*Researches and Observations on the Causes of Scrofulous Diseases.* By J. G. A. Lugol, &c.—*Paris*, 1844. 8vo, pp. 372.

